# Methanolic Extracts of *Chiococca alba* in *Aedes aegypti* Biorational Management: Larvicidal and Repellent Potential, and Selectivity against Non-Target Organisms

**DOI:** 10.3390/plants11233298

**Published:** 2022-11-29

**Authors:** Jaqueline C. M. Borges, Khalid Haddi, Wilson R. Valbon, Lara T. M. Costa, Sérgio D. Ascêncio, Gil R. Santos, Ilsamar M. Soares, Robson S. Barbosa, Kelvinson F. Viana, Eder A. P. Silva, Wellington S. Moura, Bruno S. Andrade, Eugenio E. Oliveira, Raimundo W. S. Aguiar

**Affiliations:** 1 Department of Biotechnology Biodiversity, Graduate School of Biotechnology of Amazônia (Bionorte), Federal University of Tocantins, Gurupi 77413-070, TO, Brazil; 2 Departmento de Entomologia, Universidade Federal de Lavras (UFLA), Lavras 37200-000, MG, Brazil; 3 Biotechnology Graduate Program, Federal University of Tocantins, Gurupi 77413-070, TO, Brazil; 4 Departmento de Entomologia, Universidade Federal de Viçosa (UFV), Viçosa 36570-900, MG, Brazil; 5 Department of Biology, Duke University, Durham, NC 27708, USA; 6 Natural Products Research Laboratory, Federal University of Tocantins (UFT), Palmas 77001-090, TO, Brazil; 7 Interdisciplinary Center for Life Sciences and Nature, Federal University of Latin American Integration (UNILA), Foz do Iguaçu 85870-901, PR, Brazil; 8 Department of Biological Sciences, State University of Southwest Bahia, Jequié 45206-190, BA, Brazil

**Keywords:** botanical pesticides, caninana plants, alternative insecticides, molecular docking predictions

## Abstract

The use of formulations containing botanical products for controlling insects that vector human and animal diseases has increased in recent years. Plant extracts seem to offer fewer risks to the environment and to human health without reducing the application strategy’s efficacy when compared to synthetic and conventional insecticides and repellents. Here, we evaluated the potential of extracts obtained from caninana, *Chiococca alba* (L.) Hitchc. (Rubiaceae), plants as a tool to be integrated into the management of *Aedes aegypti*, one of the principal vectors for the transmission of arborviruses in humans. We assessed the larvicidal and repellence performance against adult mosquitoes and evaluated the potential undesired effects of the extracts on non-target organisms. We assessed the susceptibility and predatory abilities of the nymphs of *Belostoma anurum*, a naturally occurring mosquito larva predator, and evaluated the *C. alba* extract’s cytotoxic effects in mammalian cell lines. Our chromatographic analysis revealed 18 compounds, including rutin, naringin, myricetin, morin, and quercetin. The methanolic extracts of *C. alba* showed larvicidal (LC_50_ = 82 (72–94) mg/mL) activity without killing or affecting the abilities of *B. anurum* to prey upon mosquito larvae. Our in silico predictions revealed the molecular interactions between rutin and the AeagOBP1 receptor to be one possible mechanism for the repellent potential recorded for formulations containing *C. alba* extracts. Low cytotoxicity against mammalian cell lines reinforces the selectivity of *C. alba* extracts. Collectively, our findings highlight the potential of *C. alba* and one of its constituents (rutin) as alternative tools to be integrated into the management of *A. aegypti* mosquitoes.

## 1. Introduction

Plants and their derivatives have long been used in folk medicine. More recently, plant extracts and their compounds have been intensively screened for insecticidal and repellent properties [1,2]. Thus, the potential uses of phytochemicals, as natural molecules and lead compounds with a wide range of activities [3,4,5], have been investigated for the control of many insect pests and disease vectors, including mosquitoes [6,7,8,9,10], although with inconsistent results. These natural insecticides are generally pest-specific, biodegradable, usually non-allergenic to humans, and less harmful to non-target organisms [8].

In recent decades, intensive research efforts have been conducted to develop natural insecticidal and repellent formulations for the control of the yellow fever mosquito, *Aedes aegypti* (Linnaeus, 1792) (Diptera: Culicidae). This mosquito is considered the principal vector responsible for the transmission of many viral diseases [11], and its control is achieved mainly through chemical products [12]. However, the reduced selectivity of the chemicals used and their adverse effects on the environment and human health, in addition to the occurrence of resistance in populations of *A. aegypti* [13,14,15], has urged the exploration of plant products as an excellent reservoir of molecules with potential ovicidal, larvicidal, pupicidal, and adulticidal effects on *A. aegypti* [16].

Along with chemical control, the use of insect repellents is an alternative strategy against mosquitoes [17]. Repellents are natural or synthetic substances that, when applied to skin, clothing, bed nets, or when emanated by vapor-emitting devices (i.e., spatial repellency) prevent mosquitoes from landing and, consequently, biting [18,19,20,21]. The best-known repellent on the market is N,N-diethyl-3-methylbenzamide (DEET) [22]. However, previous studies have reported a number of side effects of DEET, including urticarial syndrome, anaphylaxis, hypotension, and neurotoxicity [23]. Therefore, further research has been carried out to develop natural insect-repellent formulations against *A. aegypti*. Plant extracts, phytochemicals, and their formulations can serve as good repellents, with minimal effects on the environment [24].

The Neotropical region exhibits a diverse flora that remains largely underexploited as a source of biologically active substances. Naturally occurring plant species from the Brazilian Amazon savannas that are commonly used by traditional communities can provide potential avenues for developing new products against mosquitoes. *Chiococca alba* (L.) Hitachc. (Rubiacea) is a tropical plant species distributed in central and southern Brazil, Peru, French Guyana, Central America, and northward to southern Florida [25]. Known locally as cipó-cruzeiro cainca, cainana, caninana, cipó-cruz, purga-preta, raiz-preta, raiz-fedorenta, and dambre [26], *C. alba* has frequently been used in folk medicine [27,28,29]. However, little is known about the repellent and insecticidal activity of the phytochemical constituents of *C. alba* against mosquitoes.

In the present study, we prepared and characterized extracts of *C. alba* plant roots and evaluated their effects against the mosquito *A. aegypti*, as well as its selectivity toward the water bug *Belostoma anurum* (Herrich-Schäffer, 1843) (Heteroptera: Belostomatidae), a naturally occurring aquatic insect predator that is able to feed upon *A. aegypti* larva [30]. We also investigated the interaction between the principal compounds of these root extracts and the *A. aegypti* odorant-binding protein (AaegOBP1) receptor. Finally, we explored the potential repellent ability of these extracts when incorporated into gel, cream, alcohol (70%), and propyleneglycol formulations, with the aim of developing a commercially available repellent.

## 2. Material and Methods

### 2.1. Plant and Root Extracts

The plant material was derived from *C. alba* (L.) trees planted in Sucupira, Tocantins, Brazil (−11°90′51″ S; −48.85′11″ W). Roots and branches containing the leaves and flowers of *C. alba* trees were collected and taxonomic identification was performed at the herbarium of the Department of Environmental Studies of the Federal University of Tocantins (Campus Porto Nacional), where a specimen voucher was deposited under the code HTO-11.160. The research was authorized by the Sistema Nacional de Gestão do Patrimônio Genético e do Conhecimento Tradicional Associado (SISGEN) (no. AFC78EC).

Plant roots were milled using Willey-type knives. After pulverization, the material (500 g) was extracted using a Soxhlet extractor (MA-487/6/25; Marconi, Piracicaba, SP, Brazil) using methanol solvent to obtain the methanol extract (ME) at 50 °C to 75 °C for 4–6 h. The filtered extract solvent was evaporated at low pressure in a rotary evaporator (MA120; Marconi) and the resultant crude ME slurry was thoroughly dried and weighed. The collected extracts were freeze-dried at −20 °C and stored at 4 °C until further use [31].

### 2.2. Chemical Characterization of Chiococca alba Root Extract

High-performance liquid chromatography (HPLC) analyses of the ME were performed using an HPLC system (Shimadzu, Tokyo, Japan). Extract solutions and standards were prepared with methanol and filtered through Millipore (0.22-μm pore size) membranes. Separation was carried out using a gradient system fitted with a reverse-phase Phenomenex Luna 5 µm C18 (2) (250 × 4.6 mm^2^) column, with direct-connect C18 Phenomenex Security Guard Cartridges (4 × 3.0 mm^2^) filled with material similar to that of the main column. Mobile phase A was 0.1% phosphoric acid in Milli-Q water, and mobile phase B was 0.1% phosphoric acid in Milli-Q water/acetonitrile/methanol (54:35:11). Program gradient: 0 to 0.01 min, 0% B; 0.01–5 min, 0% B; 5–10 min, 30% B; 10–20 min, 40% B; 20–29 min, 40% B; 29–30 min, 50% B; 30–50 min, 100% B; 50–80 min, 100% B. The flow rate was 0.6 mL/min, and the temperature was set at 40 °C. UV detection was performed at a wavelength of 280 nm. The compounds were identified by comparing the retention times of the samples with authentic standards, including rutin, naringin, myricetin, morin, and quercetin (Sigma–Aldrich^®^, São Paulo, SP, Brazil). The quantities of the compounds were expressed in micrograms per milligram of extract (μg/mg) by correlating the area of the analyte with the calibration curve of standards built at concentrations of 4.5–18 μg/mL.

Gas chromatography and spectrometry were performed at the Analytical Center of the Institute of Chemistry of the University of São Paulo (IQUSP), using a gas chromatograph (QP5050A; Shimadzu) coupled to a mass spectrometer (GC/MS). The separation of target analytes was achieved on a DB-1 capillary column (30 m × 0.25 mm i.d., 0.25-μm film thickness). The conditions of analysis were as follows: helium was used as the drag gas, with a flow of 2.5 mL/min; programming was from 50 °C to 280 °C at 60 °C/min, with the injector and interface at 280 °C; the mass spectrometer operated with an electronic impact ionization of 70 eV and swept from 50 to 700 U [32]. The identification of the compounds was performed, based on comparisons with the library spectral databases of the National Institute of Standards and Technology (NIST). The spectrum of each of the unknown components was compared with the spectra of known components stored in the NIST library. The structures of the components in the test materials were identified based on their molecular weights.

### 2.3. Insect Populations

#### 2.3.1. *Aedes aegypti*

The population of *A. aegypti* mosquitoes was reared in the Entomology Laboratory of the Federal University of Tocantins, Campus Gurupi, according to the methodology used by the authors of [33]. Adult mosquitoes were maintained with a 10% aqueous sucrose solution and the blood of live Wistar rats (*Rattus norvegicus albinus*). The larvae were reared in plastic containers (35 × 5 cm) and fed upon turtle food (Reptolife, Alcon Pet, Camburiú, SC, Brazil). All bioassays were conducted at 26 ± 1 °C, 60.0 ± 5% RH, with a 12 h light-dark photoperiod. All applicable international, national, and institutional guidelines for the care and use of animals were considered.

#### 2.3.2. *Belostoma anurum*

Second-instar nymphs of *Belostoma anurum* (Heteroptera: Belostomatidae) were used for selectivity and predation bioassays. Nymphs of *B. anurum* were collected from a stock population reared at the Brazilian Invertebrate Neurobiology and Physiology (BraIN & Phy) Laboratory (Departamento de Entomologia) of the Universidade Federal de Viçosa (Viçosa, MG, Brazil) (20°45″ S, 42°52″ W). Briefly, nymphs (1st to 3rd instar) were kept individually in glass vials with 30 mL of dechlorinated water to avoid cannibalism and were fed daily with 4th instar *A. aegypti* (L4) larvae. Among these, 4th and 5th instar nymphs were kept individually in glass vials containing 50 mL of distilled water. Water hyacinths, *Eichhornia crassipes*, were used as resting substrates. Nymphs (4th and 5th instars) and adults of *B. anurum* were fed with adults and nymphs of backswimmers (Hemiptera: Notonectidae). The vials were placed under controlled temperature (25 ± 3 °C) and photoperiod (12:12 L:D) conditions, as previously described [30].

### 2.4. Toxicity of Chiococca alba Root Extract against Aedes aegypti Larvae and Selectivity against Its Predator, Belostoma anurum

Standard methods for assaying larvicidal activity, as recommended by the authors of [29], were followed in all experiments with ME against the 3rd instar larvae of *A. aegypti*. Briefly, a stock solution (1000 µg/mL) of each extract was prepared with 0.5% dimethyl sulfoxide (DMSO) and then diluted with dechlorinated water to obtain the desired concentrations. The ME concentrations that were used initially for the selective test were 333.3, 166.7, and 33.3 μg/mL, denoted as being strongly concentrated, intermediate, and weak, respectively. Each extract (0.1 g) was weighed and solubilized in 500 μL of DMSO and then diluted with 29.5 mL of distilled water in 200 mL disposable cups. Assays were performed in triplicate, using 25 larvae per replicate. Mortality was verified after 24 h of exposure of the 3rd instar larvae to the extracts. 

The selectivity bioassay was performed to investigate the potential side effects of the root extracts of *C. alba* on second-instar nymphs of *B. anurum.* We evaluated the susceptibility of nymphs to the *C. alba* extract and assessed whether nymphs that were sublethally exposed to the extracts would show any alteration to their abilities to prey upon *A. aegypti* larvae.

In the susceptibility bioassays, the methanolic extract was used at a concentration of 200 µg/mL, which corresponded to the LC_80_ previously estimated for mosquito larvae. The solutions were prepared from an initial concentration, with 0.1 g of extract diluted in 500 µL of DMSO. The exposure procedures followed those previously described elsewhere [34]. Briefly, nymphs (<72 h after ecdysis) were individually exposed to extracts in 15 mL glass vials containing 10 mL of extract solution. The vials were covered with organza tissue to prevent the insects from escaping. We used three replicates, with groups of 10 *B. anurum* nymphs as our experimental unit (i.e., replicates). Mortality was assessed 24 h after exposure, and the insects that remained motionless after being repeatedly stimulated mechanically with a pipette were considered dead. In the control treatment, *B. anurum* nymphs were exposed to DSMO at the proportional concentrations present in the extract solution (i.e., 0.06 μL/L).

In the predation bioassays, nymphs of *B. anurum* that survived extract exposure in the selectivity bioassays were immediately transferred and acclimated (1 h) in 200 mL glass vials containing 100 mL of distilled water (i.e., without recovery). After this acclimation period, we recorded the number of larvae preyed upon by *B. anurum* nymphs that were individually exposed to six *A. aegypti* larvae (i.e., 6 larvae/100 mL water). The number of preyed larvae was evaluated at 40-min intervals for the next 6 h, and the number of *A. aegypti* was reestablished at each evaluation in order to keep the prey density as constant as possible, following the Holling functional response experiment [35,36,37,38]. Using the same *B. anurum* nymphs, we recorded the number of larvae preyed over a 6 h period, four days after extract exposure (i.e., with 72 h of recovery). Fifteen replicates of *B. anurum* nymphs were used for each treatment (i.e., control and methanolic extract).

### 2.5. Repellency of the Pure and Formulated Extract of Chiococca alba and Cytotoxic Effects on Mammalian Cell Lines

#### 2.5.1. The Gel and Cream Formulations of the *Chiococca alba* Root Extract

The gel and cream formulations of *C. alba* root extracts were prepared as described by the authors of [39]. The formulations consisted of a gel material of anionic character (anionic 1% carboxyvinyl polymer) and a cream material, composed of O/A (oil in water), also of anionic character, with a colloidal dispersion of fatty alcohols and cetylstearyl sulfate. Two commercially available bases, Emusolut C^®^ (anionic water-cream) and Allifeel A^®^ (gel), were used as vehicles for the repellent formulations. The bases were obtained from the Fagron Laboratory, São Paulo.

#### 2.5.2. Repellency Bioassays

The repellency bioassay was performed according to the method described by the World Health Organization [11]. Four human volunteer subjects were used to test each ME formulation; the research protocol was approved by the UNIFAP Human Ethics Committee (no. 81727617.3.0000.0003). The cream and gel formulations were compared with ME in 70% alcohol (used as a vehicle). The repellents were organized into the following groups: ME, ME with gel (G), ME with cream (C), ME with propylene glycol and 70% alcohol, and ME with 70% alcohol. DEET was used as a positive control. Only 25 cm^2^ of the dorsal side of the skin on each arm of the volunteer was exposed, and the remaining area was covered with rubber gloves. The volunteers had no previous contact with lotions, perfumes, oils, or perfumed soaps on the day of the assay, which was carried out from 08:00 h to 11:00 h in a net cage.

The repellents (ME, ME with gel, ME with cream, ME with propylene glycol and 70% alcohol, ME with 70% alcohol, and DEET) were applied to the exposed area of the forearm at a concentration of 4 mg/cm^2^. The control and treated arms were introduced simultaneously into the mosquito cages (45 × 30 × 45 cm), where 50 three-day-old, blood-starved, female *A. aegypti* were kept, for one full minute. The sides of the experimental cages were gently tapped to activate the mosquitoes. Each test was repeated three times. The number of mosquitoes landing/biting in the exposed region of the hand was recorded. The repellency percentage was calculated using the following formula:

% Repellency = [(Ta − Tb)/Ta] × 100,

where Ta is the number of mosquitoes in the control group, and Tb is the number of mosquitoes in the treated group.

Based on the in silico analysis (see the Results section), we individually tested the repellency of rutin, which is one of the major constituents of the root extract. To this end, the rutin was dissolved in propylene glycol and alcohol at 70% and tested at concentrations equivalent to 0.32 g/cm^2^ and 0.16 mg/cm^2^. The negative control groups received the same solution used to solubilize the rutin. The other treatments used in this experiment were 10% DEET (Sigma–Aldrich^®^, São Paulo, SP, Brazil), 70% alcohol (5 mg/kg) (Merck Brasil, São Paulo, SP, Brazil), and propylene glycol (100 mg/kg) (Bayer, São Paulo, SP, Brazil). The repellency bioassay followed the same protocol as that used for *C. alba* ME.

#### 2.5.3. In Silico Studies of the Interaction between *Chiococca alba* Ligand Molecules and the AaegOBP1 Receptor

The crystal structure of the odorant-binding protein of *A. aegypti* was downloaded from the Protein Data Bank (https://www.rcsb.org, accessed 22 December 2021) (PDB code: 3K1E) [40]. To select this structure, we considered the quality parameters of the experimental method, resolution, and R-value, as well as its complexing with a ligand. To check the protein structure crashes and amino acid positioning in the active site, we used Chimera 1.12. Subsequently, the protein structure was adjusted for the protonation state at pH 7.5 using the H++ tool (http://biophysics.cs.vt.edu/index.php, accessed 10 January 2022). 

Molecules (ligands) representing the major components of the *C. alba* extracts were designed using Marvin Sketch 18.10 (ChemAxon, saved in 3D mol2 format). The AaegOBP1 receptors and ligands were prepared for molecular docking using AutoDock Tools 1.5.7 [41]. First, we added hydrogen atoms to compute the protonation states, then we computed all possible bond torsions for all ligands. The second step was to generate a grid box inside the receptor, indicating the coordinates for docking the ligands in the active pocket. This grid box position was designed according to the AaegOBP1 active site described by [40] for the crystallographic structure. Third, we saved the AaegOBP1 receptor and ligands in *pdbqt* format for the docking calculations, using AutoDockVina.

In the final step, we used AutoDockVina to generate nine docking positions for each ligand interacting with the OBP active site and to return energy affinity values (kcal/mol). All docking position results were analyzed using PyMOL 2.0 [42] and Discovery Studio 4.5 [43] to select the best position for each ligand inside the AaegOBP1 receptor. For this, we considered the receptor-ligand affinity energies, ligand interactions with the amino acids from the active site, and the route mean square deviation (RMSD) between the initial and subsequent ligand structures. Finally, we verified all hydrogen bonds and non-covalent interactions for each complex, according to the two-dimensional (2D) interaction maps [44].

#### 2.5.4. Cytotoxicity of the Root Extract of *Chiococca alba* and Rutin

The cytotoxicity test was carried out with TPH-1 cells (ATCC^®^ TIB-202™) from the Federal University of Latin American Integration (UNILA) stock (Paraná, Brazil) exposed to the root extract of *C. alba*, and rutin was found to be one of the major constituents. The extract and rutin were dissolved in DMSO and diluted in RPMI culture medium (Sigma–Aldrich^®^, São Paulo, SP, Brazil) to prepare a stock solution [44]. Once attached, the culture medium was removed and sample solutions were added at concentrations of 0.87, 1.30, 1.70, and 2.12 μg/mL. The final volume in each well was 100 µL, and the number of cells present in each well was 1 × 10^4^ cells. The plates were incubated for 48 h at 37 °C in a humidified atmosphere containing 5% CO_2_. Next, 100 μL of MTT (3-(4,5-dimethylthiazol-2-yl)-2,5-diphenyltetrazolium bromide) was added and incubated for 4 h. The absorbance at 540 nm was measured using a microplate spectrophotometer (Quimis^®^, Diadema, SP, Brazil). The assays were performed in triplicate.

### 2.6. Statistical Analysis

The graphs showing the repellence activity and the number of bites for *C. alba* extract and rutin with different formulations were plotted using nonlinear regression parameters, determined by OriginPro^®^ 8 software (OriginLab corporation, Northampton, MA, USA). The selectivity bioassay data were subjected to Student’s *t*-test using Sigma Plot 12.0. The number of preyed larvae obtained in the predatory bioassays was subjected to a repeated measures analysis of variance (ANOVA) to determine the effects of extract exposure and time. The number of preyed larvae at 40-min intervals on the first and fourth days after exposure was used as replicates (within-sample variation) to avoid the problems of temporal pseudo-replication [37]. The assumptions of normality and homogeneity of variance were checked using the UNIVARIATE procedure [45], and no data transformations were necessary. The total number of preyed larvae after exposure to each extract and day (i.e., 24 and 96 h after exposure) was subjected to an analysis of variance (ANOVA). A two-way RM ANOVA was used with Dunnett’s multiple comparisons against a control treatment. 

## 3. Results

### 3.1. HPLC Fingerprinting and GC-MS Analysis

**Figure 1:**
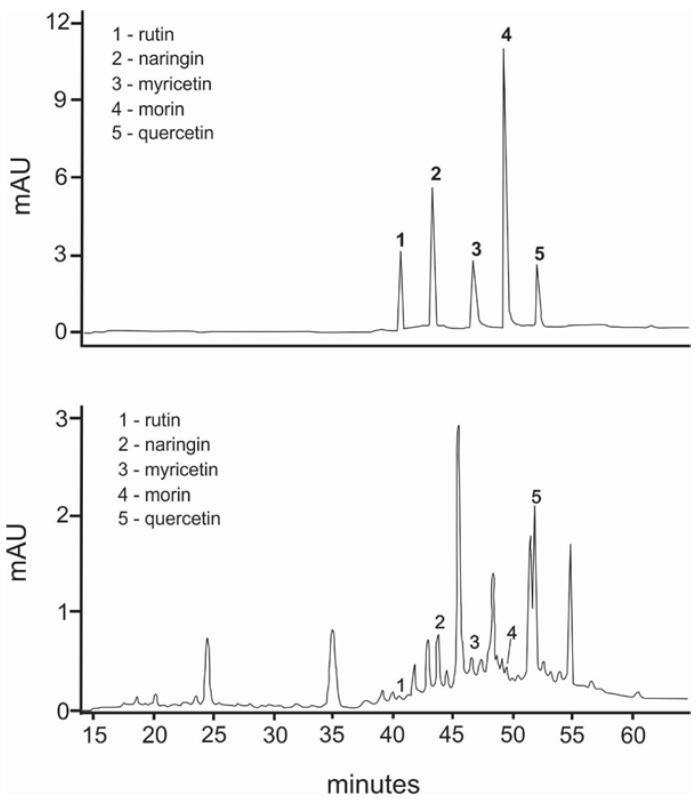
HPLC fingerprint of the methanolic extract of *Chiococca alba*, detected at 280 nm. Peak 1: rutin; peak 2: naringin; peak 3: myricetin; peak 4: morin; peak 5: quercetin. Insert: HPLC chromatogram of the authentic standards of the phenolic compounds mixture.

Chromatographic profiles (fingerprints) obtained by HPLC of the ME of *C. alba* roots are shown in Figure 1. The data show the presence of rutin, naringin, myricetin, morin, and quercetin at the respective concentrations: 1.86 μg/mg (rutin), 8.96 μg/mg (naringin), 8.60 μg/mg (myricetin), 2.54 μg/mg (morin), and 8.31 μg/mg (quercetin). The results of the GC-MS analysis of the ME of C. alba are given in Table 1. Eight compounds were identified from the retention time (RT) and mass data, and by comparing the data of the standard compounds with those in the library and literature. Some compounds remained unidentified, owing to the lack of reference substances and library spectra. A total of 20 peaks were isolated, and 14 compounds were identified using NIST-2005. As shown in Table 1, phytosterol and saturated fatty acids constituted much of the *C. alba* methanolic extract. The major components identified in the extract of *C. alba* were palmitic acid (10.83%), 3,5-methoxycinnamic acid (5.49%), coniferol (2.98%), 1-(2-hydroxyphenyl) ethanone (2.61%), tetradecanoic acid (1.81%), and 1,6-anhydro-beta-D-glucopyranose (3.56%).

### 3.2. Toxicity of Chiococca alba Extracts to Aedes aegypti Larvae and Belostoma anurum Nymphs

**Figure 2:**
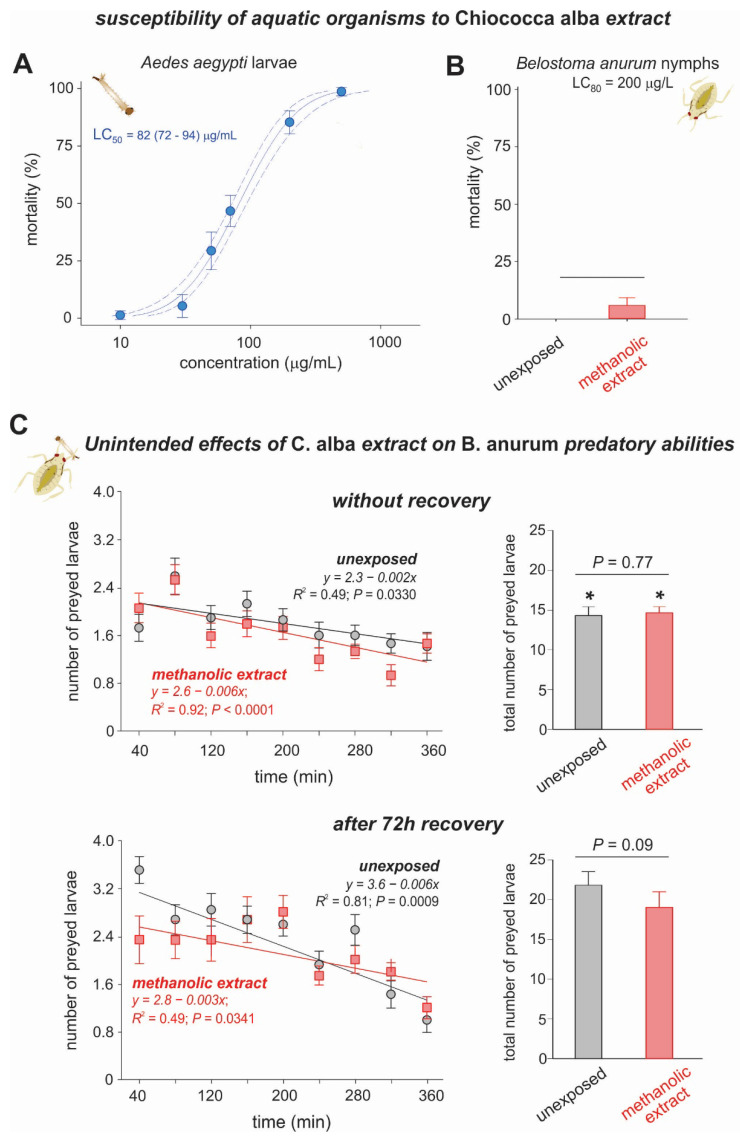
Susceptibility of *Aedes aegypti* larvae (**A**) and *Belostoma anurum* nymphs (**B**) to methanolic extracts of *Chiococca alba*. (**C**) The number of *A. aegypti* larvae preyed upon by *B. anurum* nymphs over time, with and without a period (96 h) of recovery after exposure to the *C. alba* methanolic extract (200 µg/mL) (**left**). The total number of *A. aegypti* larvae preyed upon by *B. anurum* nymphs (**right**). The control treatment consisted of unexposed nymphs. (**A**) A filled circle indicates the mortality values obtained with the extract application, while dotted lines represent the 95% confidence intervals. (**B**) Data are the mean ± SE. (**C**) The predatory ability was assessed at the larval density of six larvae/100 mL of water. Larval densities were reestablished after every evaluation. Symbols show the average number of larvae preyed upon by each *B. anurum* nymph (*n* = 15). Data are the mean ± SE. * denotes significant differences in the total number of preyed larvae between the two exposure times (i.e., without recovery and after 72 h recovery).

The mortality data obtained for the methanolic extracts of *C. alba* against mosquito larvae fit the probit model (χ2 = 1.1; *p* = 0.36) and resulted in an LC_50_ of 82 (72–94) μg/mL (Figure 2A). The results for *B. anurum* nymphs showed that the *C. alba* methanolic extracts did not kill more than 10% of these aquatic predator insects, showing no difference compared to the unexposed *B. anurum* nymphs (*t* = −1.0, df = 4, *P* = 0.374), even when applied at 200 μg/mL; that is, the maximum estimated value for the LC_80_ (161 (137–200) μg/mL) obtained for the *A. aegypti* larvae (Figure 2B).

#### Effects of *Chiococca alba* Extract on the Predatory Abilities of *Belostoma anurum*

To assess whether exposure to *C. alba* extracts reduced the predatory abilities of *B. anurum* nymphs, we exposed the predators to methanolic extract (at a concentration of 200 µg/mL). The analysis of variance with repeated measures over time for the number of *A. aegypti* larvae preyed upon at 40-minute intervals for 24 h (i.e., without recovery) of exposure showed significant effects, although only for duration and for its interaction with the extract (Figure 2C, Table 2). After 96 h (i.e., 72 h of recovery) of extract exposure, however, a significant effect was observed, although only over time (Figure 2C left, Table 3). The total number of preyed larvae was not affected by either extract exposure or the interaction between extract and time; however, a significant effect was observed for recovery time (Figure 2C, right, and Supplementary Table S2). Although there was no extract effect, the total number of prey larvae for *B. anurum* after recovery was higher, not only for unexposed (control) but also for extract-exposed insects (two-way RM ANOVA: treatment *F*_(1,14)_ = 0.501, *p* = 0.491; time recovery *F*_(1,14)_ = 67.886, *p* < 0.001; treatment vs. time recovery *F*_(1,22)_ = 1.917, *p* = 0.188; Figure 2C, right). The nymphs of *B. anurum* showed a consumption of 14.3 ± 0.25 (24 h after exposure) and 20.6 ± 0.79 (96 h after exposure) (Figure 2C, right).

### 3.3. Repellent Activity of Formulations Containing an Extract of Chiococca alba and Rutin

**Figure 3:**
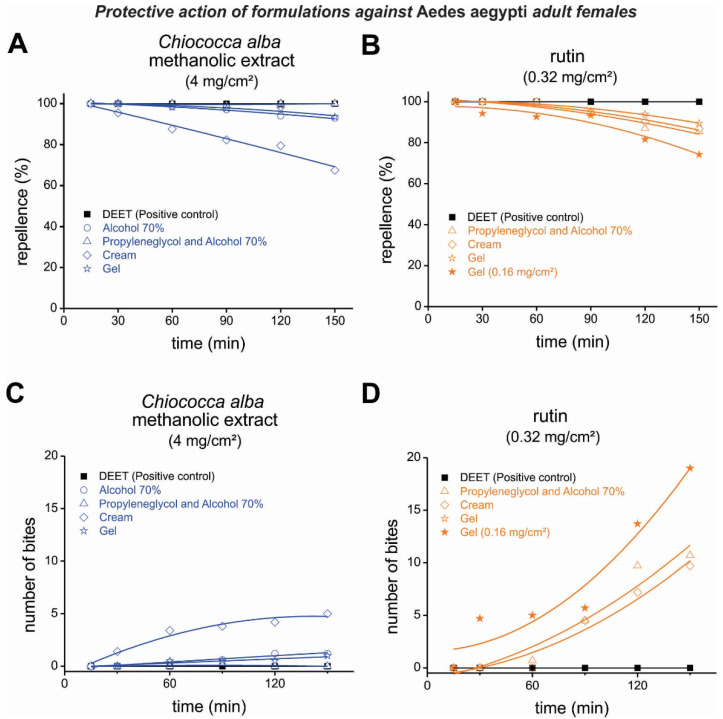
Repellent activity of formulations containing the *Chiococca alba* methanolic extract (**A**) and rutin (**B**) against *Aedes aegypti* adult females. (**C**,**D**) The number of bites from *A. aegypti* females after the application of *C. alba* methanolic extract (**C**) and rutin (**D**) in different formulations on human volunteers’ forearms. The parameters of the nonlinear regressions are described in Supplementary Table S1.

The gel formulation of the methanolic extract (4 mg/cm^2^) protected the exposed arms for 120 min, while the cream formulation showed a protection period of 90 min; although it decreased over time, it continued above 60% for up to 150 min. Similarly, ME and alcohol promoted repellency for 90 min. These results compare favorably with those of the positive control DEET 10%, exhibiting 100% repellency (Figure 3A,C). The ME of *C. alba* and propylene glycol with 70% alcohol protected the skin of the exposed arms for up to 150 min, while propylene glycol and 70% alcohol alone had no repellent effect.

When tested alone, rutin at a concentration of 0.32 mg/cm^2^ demonstrated up to 100% protective activity in the screening bioassay for 30 min for all tested formulations. In addition, gel-formulated rutin (0.32 mg/cm^2^) provided 89.5% protective activity during the whole period of the test (Figure 3B,D).

#### Interaction between the Molecules of *Chiococca alba* and the AeagOBP1 Receptor of *Aedes aegypti*

After the molecular docking calculations, the affinity energy values were obtained for all compounds tested, as shown in Table 4. The compounds were ranked from the best to the worst affinity energy, with greater negative values representing stronger interactions. The ligands of rutin, oleyl alcohol trifluoroacetate, and 3,5-dimethoxycinnamic acid showed higher affinity energies, as well as better positioning and fit within the active site of AeagOBP1. Detailed descriptions of the mechanisms by which these molecules interact with AeagOBP1 are provided below.

**Figure 4:**
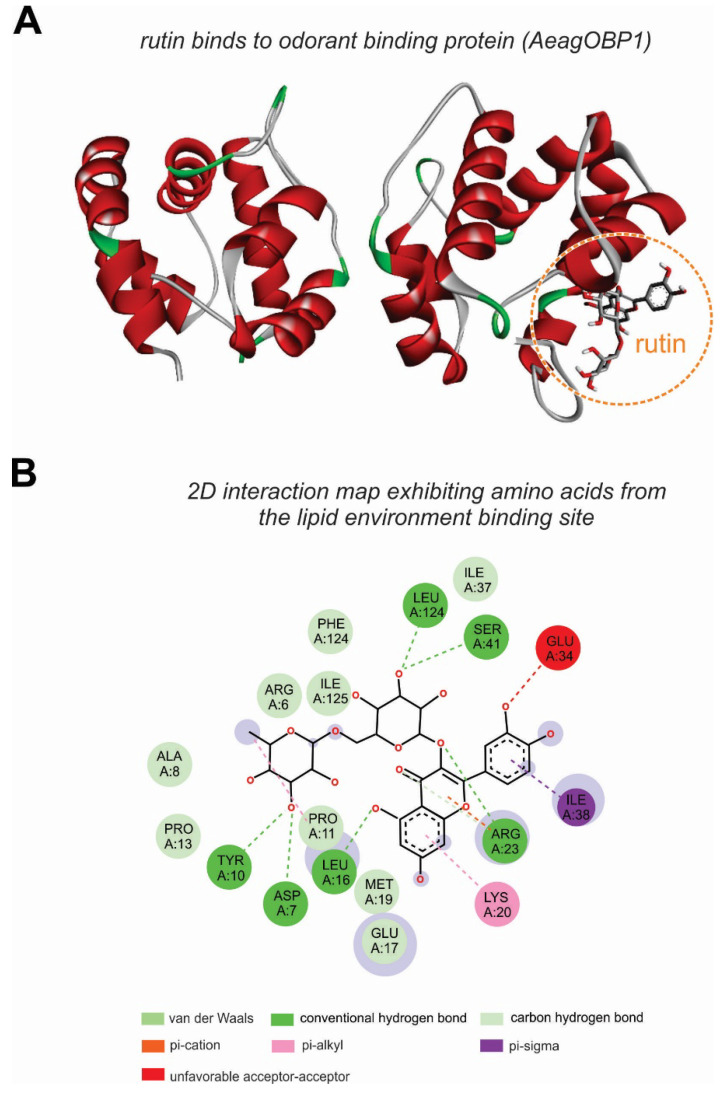
Rutin binds to the *Aedes aegypti* odorant binding protein (AeagOBP1). (**A**) Docking view of rutin with the AeagOBP1 binding site. (**B**) The 2D maps demonstrate the molecular interactions with amino acids from the AeagOBP1active site and rutin.

The rutin molecule presented a surface area larger than that of the OBP binding cavity, which makes it difficult to position it within the binding pocket of the receptor. Thus, it uses molecular anchoring to interact with a cavity close to the important amino acids of the receptor, such as Arg23 and Ile125, while remaining outside the binding pocket (Figure 4A); however, it formed several electrostatic bonds and, thus, had considerable affinity energy (−8.1 kcal/mol). Figure 4B shows rutin complexed with OBP, positioned out of the binding pocket, and 2D maps of the molecular interactions with amino acids in the active site of the *A. aegypti* receptor. This very complex structure, with diverse aromatic rings and oxygens, favors interactions with high stability because of the forces involved. The map indicates that six hydrogen bonds were formed with the residues Tyr10, Asp7, Leu16, Arg23, Ser41, and Leu124. Some of the hydrophobic, Pi-alkyl, alkyl, Pi-sigma, and Pi-cation characteristics also formed weaker interactions (van der Waals), due to the distance between the molecule and other amino acid residues (Arg6, Ala8, Pro 11, Pro13, Glu17, Met19, Phe123, and Ile125).

The 3,5-dimethoxycinnamic acid compound had an affinity energy for binding to the *A. aegypti* mosquito receptor (AeagOBP1 code PDB: 3K1E) of −7.0 kcal/mol, forming van der Waals interactions with the residues of Phe15, Leu58, Phe59, Val64, Phe123, and Ile125, and P1-alkyl interactions of the aromatic ring of the linker with the alkyl groups of the residues Leu76 and Leu80. In addition, the oxygen of the Ala88 carboxyl group formed a hydrogen bond at a distance of 3.56 Å, which is characteristic of a strong bond. These interactions are responsible for the specificity of molecular recognition and intensity, thus demonstrating the stability of the compound’s bond to the receptor’s principal amino acid residues in its binding well. Other amino acids (aa) were not observed in the binding pocket but provide additional interactions for the stability of the compound binding to the receptor, such as the residues Met91, Trp114, and His121, with interactions via the Pi-sulfur, Pi-sigma, and hydrogen bonds. These are considered strong interactions, which contributed to the high stability of the compound’s overall interaction with the receptor (Supplementary Figure S1).

The oleoyl alcohol trifluoroacetate compound has a long-chain carbon structure, with a polarized, oxygen-containing molecule (which has free electronic pairs and a high electronic density) and a set of three (3) fluorine atoms, which are very dense electrostatically. The interactions between the residues and the molecule are based on the polar part of the molecule, with halogen bonds forming to the Ala62 and Ala79 oxygen atoms, as well as the apolar part at the beginning of the carbon chain, with alkyl interactions of the ligand with the amino acids Ala88, Leu76, and Leu80. There are also Pi-alkyl interactions with the aromatic ring of Trp114 and with other amino acids (Phe15, Met19, Leu58, Phe59, Val64, Met84, Met91, Gly94, His121, Leu124, and Ile125) via van der Waals forces, forming a large number of interactions that increase ligand–receptor interaction, thus exhibiting an affinity energy of −7.4 kcal/mol (Supplementary Figure S2).

### 3.4. Cytotoxicity of the Root Extract of Chiococca alba and Rutin to THP-1 Cells

**Figure 5:**
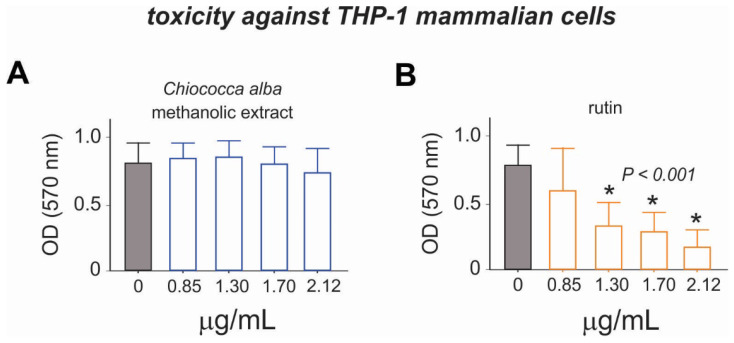
Toxicity of the methanolic extract of *Chiococca alba* and rutin to the human monocyte, THP-1. (**A**) The MTT (3-(4,5-dimethylthiazol-2-yl)-2,5-diphenyltetrazolium bromide) test after 48 h of cell exposure to the *C. alba* extracts, indicating cell death at concentrations of 0.85, 1.30, 1.70, and 2.12 μg/mL (*P* < 0.0001). (**B**) The MTT test after 48 h of cell exposure to the rutin, indicating cell death at concentrations of 1.30, 1.70, and 2.12 μg/mL (* Significant at *p* < 0.001).

The cytotoxicity results indicated that ME presented low toxicity, while rutin presented a high degree of toxicity in the cell line, especially at concentrations of 1.30, 1.70, and 2.12 μg/mL by the MTT test (*p* < 0.001; Figure 5).

## 4. Discussion

In the present study, we used methanol as a solvent to prepare the *C. alba* extract and its cream and gel formulations. The extract was subsequently characterized and tested for its toxic activity against mosquitoes, as well as its selectivity toward a non-target organism, while its cream and gel formulations were assessed for their repellency to adult mosquitoes. Furthermore, we modeled the interactions between the major chemical compounds identified in ME, with the AeagOBP1 receptor of *A. aegypti*. 

Methanol is a good solvent for the extraction of polyphenolic compounds, as its polarity promotes the extraction of aromatic compounds that contain hydroxyls [46]. Here, the five major compounds present in the ME of *C. alba* were rutin, narigin, myricetin, morin, and quercetin. However, the components of the *C. alba* extracts may vary as a result of different ecological conditions, such as the type of soil, climate, degree of maturity, and physiological development of the plant [47]. Phytochemical constituents, such as lignans, coumarins, ketoalcohols [48], triterpenes, glucuronide glycosides [25], iridoids [49], nor-seco-pimarane [50], two quinoline alkaloids [51], and flavonoids [52] have also been previously identified after the methanolic extraction of *C. alba*.

Herein, the ME of *C. alba* exhibited insecticidal toxicity toward *A. aegypti* larvae. Plant extracts have been shown to exert insecticidal or repellent activity against several insect species, including mosquitoes. Plant extracts usually contain alkaloids, saponins, terpenoids, and tannins, which have insecticidal properties against insect vectors [53]. Although the mode of action of *C. alba* extract has not been previously investigated, its toxic effects may be derived from the actions of its major components. Rutin, naringin, morin, and quercetin have been identified in *C. alba* roots and have been shown to have toxic effects against *A. aegypti* larvae [54,55,56,57]. Larval mortality was reported to be between 44% and 100% (48 h) for quercetin nanosuspensions at 100 and 500 ppm, respectively [58]. Naringin, a phenolic compound, showed toxic activity against *A. aegypti* larvae at low concentrations and deterred oviposition effects [54]. Morin inhibits the compliance-like mitochondrial transhydrogenase activity in *Manduca sexta* larvae [55].

Our results also indicate that the ME of *C. alba* roots showed repellent effects against *A. aegypti* adult mosquitoes. The increase in the protection time at higher concentrations may be due to an increase in the concentration of the active ingredients present in the extracts. Phytochemicals obtained from the different plant species are important sources of safe and biodegradable chemicals that can be screened for mosquito-repellent and insecticidal activities. Repellents of plant origin do not pose toxicity hazards to humans or domestic animals and are easily biodegradable [59]. Repellents are substances that act locally or at a distance, deterring an insect from flying to, landing on, or biting human or animal skin (or a surface in general) [60]. Various ME formulations of *C. alba* were evaluated in this study. The mosquito bite deterrent effect of ME and 70% alcohol was very promising for topical use; the preparation can be reapplied if a longer effect is required, provided that no adverse effects are produced. We chose the extracts of the *C. alba* formulations in non-ionic creams and hydrophilic gels for their scatter ability, good active permeation, and low oiliness. Formulations prepared with propylene glycol and 70% alcohol were added to the extract, to allow fixation on the human skin of volatile components present in the ME, in addition to effective absorption, good drying, and texture on the skin [61].

Tests performed with the non-target organism *B. anurum,* which is a naturally occurring predator of mosquitoes, demonstrated the selectivity of the extract for this organism, favoring its action against the target organism [16]. In addition, the *C. alba* extract showed no cytopathic effects on TPH-1 human cells, indicating that *C. alba* extract could be safely used as a repellent agent against this mosquito. Although the major component, rutin, presented some cytotoxic effects at high concentrations (beyond 1.30 µg/mL), very good repellent activity was achieved with a gel formulation at 0.32 mg/cm^2^, reinforcing the potential of this substance as a mosquito repellent.

The repellence mechanism of action of the *C. alba* extract against *A. aegypti* could be explained by the direct interactions of its constituents with the AaegOBP1 receptor. Regarding the correlations between the binding mode and repellence, it is generally hypothesized that repellence activity is related to longer docking times for molecules interacting with the internal pocket regions, compared to other molecules in the external region [62]. In addition to DEET presenting a binding site at the center of a long hydrophobic tunnel [63], this hypothesis was also verified for another phytochemical, lapachol, which has been shown to be responsible for the repellent activity of *Tabebuia heptaphylla* extracts [6].

In our docking results with the major compounds of the *C. alba* extract, the ligands 3,5-dimethoxycinnamic acid, oleoylalcohol trifluoroacetate, and rutin showed the highest affinity energies, as well as better positioning and fit within the active site of AeagOBP1, but interacted in distinct regions of the pocket. Even though the rutin ligand did not dock inside the AeagOBP1 interior binding site because of its larger surface area, this molecule presented the best affinity energy, as well as interaction with amino acids closer to the receptor’s cavity, such as Arg23 and Ile125. This suggests that the principal role of the mechanism of action of rutin is repellency activity, as described in other insects [64,65,66].

OBPs play an important role in the insect olfactory system by transporting odorant molecules through the sensillar lymph in the antennae [67]. Our docking and behavioral findings suggest that the *A. aegypti* olfactory system is likely an important molecular target for the non-contact repellent actions of the *C. alba* extract. Spatial repellency occurs when mosquitoes encounter molecules in the vapor phase before directly contacting the treated surface, as observed in natural and synthetic compounds with both insecticidal and repellent actions [20,21,68]. However, we cannot rule out a contact repellency mechanism through the activation of different targets beyond the olfactory system [21,69,70]. Although such findings on the interaction of *C. alba* compounds with the AaegOBP1 receptor of *A. aegypti* allow for a better understanding of the repellence mechanisms, there is a need to investigate their interactions with the other types of antennal olfactory receptor neurons (ORNs) of mosquitoes. Some authors have reported that two or more compounds are responsible for modifying or blocking the responses of normally sensitive ORNs to attractants [71,72,73]. In this context, the elucidation of these mechanisms can increase the potential for the design and development of a new generation of synthetic repellents against major mosquito vectors of infectious diseases.

## 5. Conclusions

In this study, the insecticidal effects of the methanolic root extracts of *C. alba* against the mosquito larvae, together with its selectivity to a non-target natural predator and low cytotoxicity, demonstrated its potential as a control tool. Its gel formulation action against the adults provided timely protection against mosquito female bites, and the interaction of its major components with the AaegOBP1 receptor supports its prospective use as a repellent. In light of these findings, the methanolic root extracts of *C. alba* should be assessed further as a possible opportunity for the inclusion of potential molecules in mosquito control programs for both indoor and outdoor applications. 

## Figures and Tables

**Table 1:** GC–MS analysis of phytochemical compounds in the ME of *Chiococca alba*.

Peak	Constituent	Chemical Structure	Retention Time	Molecular Formula	Chemical Composition (%)
01	non-identified	-	3.069	C_14_H_22_O	4.43
02	1-Deoxy-2,4-O-methylene-D-xylitol	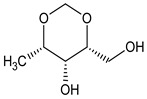	5.710	C_6_HC_12_O_4_	1.93
03	2-fenilpropanal	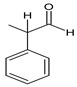	6.670	C_9_H_10_O	4.21
04	1-(2-hydroxyphenyl) ethanone	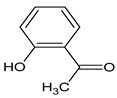	6.914	C_8_H_8_O_2_	2.61
05	2-isopropoxifenol	-	7.375	C_9_ H_12_O_2_	1.54
06	non-identified		9.026	-	6.87
07	2,6-dimethoxyphenol	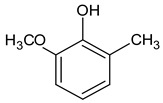	9.519	C_8_H_10_O_3_	2.32
08	3-nitrobenzyl iodide	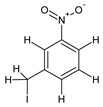	10.655	C_7_H_6_INO_2_	6.01
09	1,6-Anhydro-beta-D-glucopyranose	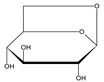	11.142	C_6_H_10_O_5_	3.56
10	non-identified	-	12.212	-	2.47
11	non-identified	-	12.646	-	10.15
12	(E)-4-Methoxycinnamic acid	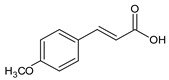	13.776	C_10_H_10_O_3_	5. 05
13	Coniferol	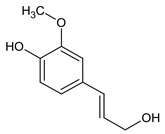	14.186	C_10_H_12_O_3_	2.98
14	Tetradecanoic acid	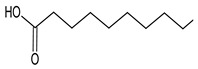	14.298	C_14_H_28_O_2_	1.81
15	3,5-Dimethoxycinnamic acid	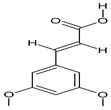	15.899	C_11_H_12_O_4_	5.49
16	Oleyl alcohol, trifluoroacetate	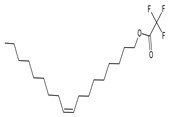	16.150	C_20_H_35_F_3_O_2_	1.55
17	Palmitic acid	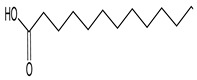	16.347	C_16_H_32_O_2_	10.83
18	non-identified	-	23.613	-	2.97
19	non-identified	-	30.302	-	11.19
20	Tris (2,4-di-terc-butilfenil)	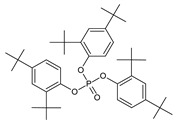	33.683	C_42_H_63_O_4_P	12.5

**Table 2:** Analysis of variance with repeated measures over time for the mean number of *Aedes aegypti* larvae preyed upon (at 40-min intervals) by *Belostoma anurum* after 24 h of exposure to extracts of *Chiococca alba* (i.e., without recovery).

Sources of Variation	*df*	*F*	*p*		
Between factors					
Extract (Ex)	1	0.06	0.80		
Error	28	-	-		
	*df_num_*	*df_den_*	Wilks’ lambda	*F*	*P*
Within each factor					
Time (T)	8	21	0.309	5.87	0.0050 *
T × Ex	8	21	0.436	3.40	0.0116 *

* Significant at *p* < 0.05.

**Table 3:** Analysis of variance with repeated measures over time for the mean number of *Aedes aegypti* larvae, preyed upon (at 40-minute intervals) by *Belostoma anurum* after 96 h of exposure to the extract of *Chiococca alba* (i.e., with 72 h of recovery).

Sources of Variation	*df*	*F*	*p*		
Between factors					
Extract (Ex)	1	3.06	0.09		
Error	28	-	-		
	*df_num_*	*df_den_*	Wilks’ lambda	*F*	*P*
Within each factor					
Time (T)	8	21	0.145	15.42	<0.0001 *
T × Ex	8	21	0.617	1.63	0.17

* Significant at *p* < 0.05.

**Table 4:** Results of the calculations and molecular coupling of molecules identified from the ME of *Chiococca alba*.

Binding	Compounds	Affinity Energy (kcal/mol)
14	Rutin	−8.1
12	Oleyl alcohol, trifluoroacetate	−7.4
11	3,5-Dimethoxycinnamic acid	−7
15	Morin	−7
8	(E)-4-Methoxycinnamic acid	−6.9
9	Coniferol	−6.8
10	Tetradecanoic acid	−6.5
6	3-nitrobenzyl iodide	−6.4
13	Palmitic Acid	−6.4
2	2-phenylpropanal	−6.1
3	1- (2-Hydroxyphenyl) ethanone	−6
4	2-isopropoxyphenol	−5.9
5	2,6-dimethoxyphenol	−5.7
7	1,6-Anhydro-beta-D-glucopyranose	−5.2
1	1-Deoxy-2,4-O-methylene-D-xylitol	−5

## Data Availability

The raw data presented in this study are available on request from the corresponding authors.

## Supplementary Materials

The following supporting information can be downloaded at: https://www.mdpi.com/article/10.3390/plants11233298/s1, Table S1. Summary of nonlinear regression analyses of the curves shown in Figure 2. Table S2. Analysis of variance with repeated measures over time for total number of *Aedes aegypti* larvae preyed (with and without recovery) by *Belostoma anurum* after exposure to extract of *Chiococca alba*. Figure S1. 3,5-dimethoxcinnamic acid binds to *A. aegypti* odorant binding protein (AeagOBP1). The 2D maps demonstrating molecular interactions with amino acids from AeagOBP1active site and 3,5-dimethoxcinnamic acid.
