# Supplementary material for: Methanolic Extracts of Chiococca alba in Aedes aegypti Biorational Management: Larvicidal and Repellent Potential, and Selectivity against Non-Target Organisms
Source: Plants (Basel). 2022 Nov 29;11(23):3298. doi: 10.3390/plants11233298 (PMC9735851; doi:10.3390/plants11233298)
Supplement: Supplementary file 1 [file plants-11-03298-s001.zip › plants-1999357-supplementary.pdf]

**Supplementary Table S1 -** Summary of nonlinear regression analyses of the curves shown in **Figure 2**.

| | | | |
|---|---|---|---|
| Model (**Figure 2A**) | Polynomial | | |
| Adj. R-Square | -- | 0,96301 | -0,135 |
| | | Value | Standard Error |
| DEET (Control) | Intercept | 100 | 1,42431E-14 |
| DEET (Control) | B1 | -7,71546E-16 | 4,31764E-16 |
| DEET (Control) | B2 | 3,76904E-18 | 2,58808E-18 |
| Alcohol 70% | Intercept | 100,75997 | 0,72075 |
| Alcohol 70% | B1 | -0,0286 | 0,02185 |
| Alcohol 70% | B2 | -1,70154E-4 | 1,30965E-4 |
| Propylene glycol and alcohol 70% | Intercept | 100,39811 | 0,54404 |
| Propylene glycol and alcohol 70% | B1 | -0,01955 | 0,01649 |
| Propylene glycol and alcohol 70% | B2 | 1,14646E-4 | 9,88564E-5 |
| Gel | Intercept | 99,89907 | 1,15496 |
| Gel | B1 | 0,00218 | 0,03501 |
| Gel | B2 | -2,73686E-4 | 2,09865E-4 |
| Cream | Intercept | 102,04376 | 3,16329 |
| Cream | B1 | -0,20373 | 0,09589 |
| Cream | B2 | -1,0315E-4 | 5,74793E-4 |
| | | | |
| Model (**Figure 2B**) | Polynomial | | |
| Adj. R-Square | -- | 0,91289 | 0,94179 |
| | | Value | Standard Error |
| DEET | Intercept | 100 | 1,42431E-14 |
| DEET | B1 | -7,71546E-16 | 4,31764E-16 |
| DEET | B2 | 3,76904E-18 | 2,58808E-18 |
| Propyleneglycol and Alcohol 70% | Intercept | 101,36842 | 2,45177 |
| Propyleneglycol and Alcohol 70% | B1 | -0,0329 | 0,07432 |
| Propyleneglycol and Alcohol 70% | B2 | -5,47212E-4 | 4,45506E-4 |
| Gel | Intercept | 100,22064 | 1,30296 |
| Gel | B1 | 0,00991 | 0,0395 |
| Gel | B2 | -5,43613E-4 | 2,36759E-4 |
| Cream | Intercept | 100,79593 | 1,75284 |
| Cream | B1 | -0,00747 | 0,05314 |
| Cream | B2 | -6,05363E-4 | 3,18504E-4 |
| Gel * | Intercept | 97,66122 | 4,16982 |
| Gel * | B1 | 0,01373 | 0,1264 |
| Gel * | B2 | -0,00113 | 7,57688E-4 |
| | | | |
| Model (**figure 2C**) | Polynomial | | |
| Adj. R-Square | -- | 0,91936 | -0,135 |
| | | Value | Standard Error |

| | | | |
|---|---|---|---|
| DEET (Control) | Intercept | 0 | 0 |
| DEET (Control) | B1 | 0 | 0 |
| DEET (Control) | B2 | 0 | 0 |
| Alcohol 70% | Intercept | -0,24169 | 0,19281 |
| Alcohol 70% | B1 | 0,01103 | 0,00584 |
| Alcohol 70% | B2 | -5,60492E-6 | 3,50359E-5 |
| Propylene glycol and alcohol 70% | Intercept | -0,07962 | 0,10881 |
| Propylene glycol and alcohol 70% | B1 | 0,00391 | 0,0033 |
| Propylene glycol and alcohol 70% | B2 | -2,29292E-5 | 1,97713E-5 |
| Gel | Intercept | -0,1 | 0,22838 |
| Gel | B1 | 0,00667 | 0,00692 |
| Gel | B2 | 1,26172E-20 | 4,14976E-5 |
| Cream | Intercept | -0,86071 | 0,57887 |
| Cream | B1 | 0,07938 | 0,01755 |
| Cream | B2 | -2,80501E-4 | 1,05184E-4 |
| | | | |
| Model (**Figure 2D**) | Polynomial | | |
| Adj. R-Square | -- | 0,9074 | 0,89754 |
| | | Value | Standard Error |
| DEET (Control) | Intercept | 0 | 0 |
| DEET (Control) | B1 | 0 | 0 |
| DEET (Control) | B2 | 0 | 0 |
| Propyleneglycol and Alcohol 70% | Intercept | -1,1513 | 1,86546 |
| Propyleneglycol and Alcohol 70% | B1 | 0,03125 | 0,05655 |
| Propyleneglycol and Alcohol 70% | B2 | 3,61517E-4 | 3,38968E-4 |
| Gel | Intercept | 1,60475 | 2,78932 |
| Gel | B1 | 1,34422E-4 | 0,08456 |
| Gel | B2 | 7,72428E-4 | 5,06841E-4 |
| Cream | Intercept | -0,71947 | 1,39449 |
| Cream | B1 | 0,01195 | 0,04227 |
| Cream | B2 | 4,05401E-4 | 2,53389E-4 |
| Gel* | Intercept | 1,60475 | 2,78932 |
| Gel* | B1 | 1,34422E-4 | 0,08456 |
| Gel* | B2 | 7,72428E-4 | 5,06841E-4 |

**Supplementary Table S2**. Analysis of variance with repeated measures over time for total number of *A. aegypti* larvae preyed (with and without recovery) by *B. anurum* after exposure to extract of *C. alba*.

| Sources of variation | *df* | *F* | *P* | | |
|---|---|---|---|---|---|
| Between factors | | | | | |
| Extract (Ex) | 1 | 3.43 | 0.07 | | |
| Error | 28 | - | - | | |
| | $df_{num}$ | $df_{den}$ | Wilks' lambda | *F* | *P* |
| Within each factor | | | | | |
| Recovery time (RT) | 1 | 28 | 0.247 | 85.57 | <0.0001* |
| RT × Ex | 1 | 28 | 0.960 | 1.17 | 0.288 |

‘

2D maps interactions with 3,5-dimethoxycinnamic acid

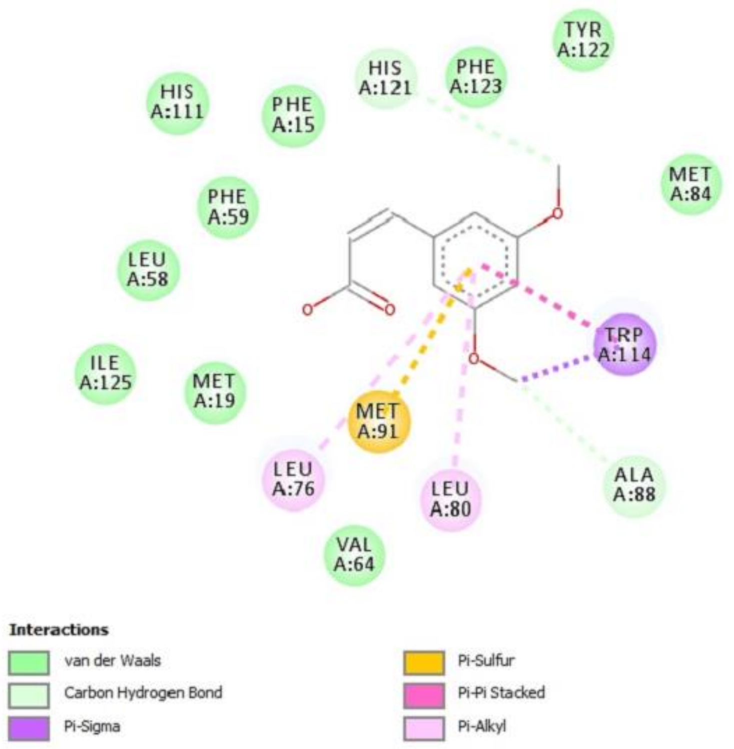


**Supplementary Figure S1**. 3,5-dimethoxcinnamic acid binds to *Aedes aegypti* odorant binding protein (AeagOBP1). The 2D maps demonstrating molecular interactions with amino acids from AeagOBP1active site and 3,5-dimethoxcinnamic acid.

## 2D maps interactions with oleoyl alcohol trifluoroacetate

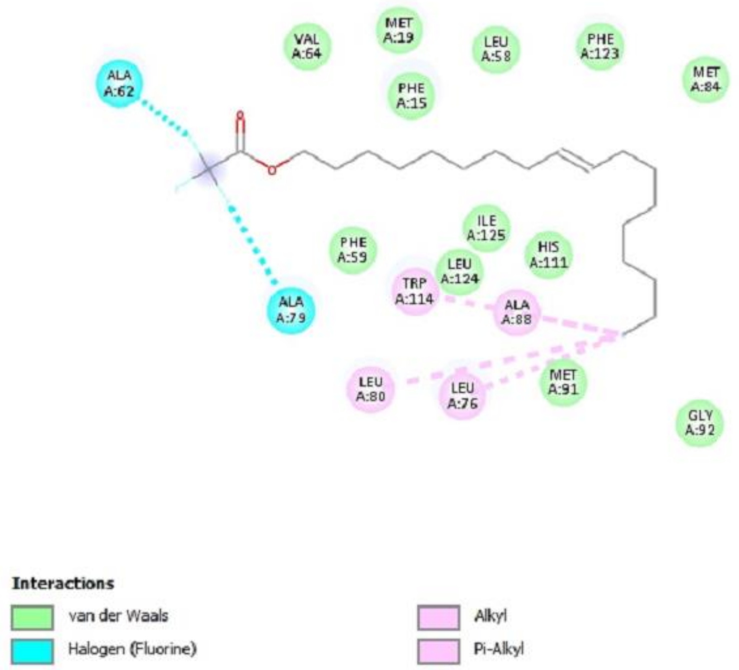


**Supplementary Figure S2.** Oleoyl alcohol trifluoroacetate binds to *Aedes aegypti* odorant binding protein (AeagOBP1). The 2D maps demonstrating molecular interactions with amino acids from AeagOBP1active site and oleoyl alcohol trifluoroacetate.
